# A Differential Inertial Wearable Device for Breathing Parameter Detection: Hardware and Firmware Development, Experimental Characterization

**DOI:** 10.3390/s22249953

**Published:** 2022-12-16

**Authors:** Roberto De Fazio, Maria Rosaria Greco, Massimo De Vittorio, Paolo Visconti

**Affiliations:** 1 Department of Innovation Engineering, University of Salento, 73100 Lecce, Italy; 2 Center for Biomolecular Nanotechnologies, Italian Institute of Technology IIT, 73010 Arnesano, Italy

**Keywords:** wearable devices, chest movements, inertial sensors, breathing parameters, digital filtering, Bland–Altman analysis

## Abstract

Breathing monitoring is crucial for evaluating a patient’s health status. The technologies commonly used to monitor respiration are costly, bulky, obtrusive, and inaccurate, mainly when the user moves. Consequently, efforts have been devoted to providing new solutions and methodologies to overcome these limitations. These methods have several uses, including healthcare monitoring, measuring athletic performance, and aiding patients with respiratory diseases, such as COPD (chronic obtrusive pulmonary disease), sleep apnea, etc. Breathing-induced chest movements can be measured noninvasively and discreetly using inertial sensors. This research work presents the development and testing of an inertia-based chest band for breathing monitoring through a differential approach. The device comprises two IMUs (inertial measurement units) placed on the patient’s chest and back to determine the differential inertial signal, carrying out information detection about the breathing activity. The chest band includes a low-power microcontroller section to acquire inertial data from the two IMUs and process them to extract the breathing parameters (i.e., RR—respiration rate; TI/TE—inhalation/exhalation time; IER—inhalation-to-exhalation time; V—flow rate), using the back IMU as a reference. A BLE transceiver wirelessly transmits the acquired breathing parameters to a mobile application. Finally, the test results demonstrate the effectiveness of the used dual-inertia solution; correlation and Bland–Altman analyses were performed on the RR measurements from the chest band and the reference, demonstrating a high correlation ($\bar{r}$ = 0.92) and low mean difference ($\bar{\mathrm{MD}}$ = −0.27 BrPM (breaths per minute)), limits of agreement ($\bar{\mathrm{LoA}}$ = +1.16/−1.75 BrPM), and mean absolute error ($\bar{\mathrm{MAE}}$ = 1.15%). Additionally, the experimental results demonstrated that the developed device correctly measured the other breathing parameters (TI, TE, IER, and V), keeping an MAE of ≤5%. The obtained results indicated that the developed chest band is a viable solution for long-term breathing monitoring, both in stationary and moving users.

## 1. Introduction

The respiration rate (RR) is a vital sign that provides information on clinical deterioration, predicts cardiac arrest, and supports the diagnosis of severe pneumonia, asthma, dehydration, fever, infections, and overdoses [1,2,3]. Furthermore, the RR responds to various stressors, including emotional stress, cognitive load, cold, and hyperthermia. Additionally, the RR is a good marker of physical effort and fatigue during exercise, and is associated with exercise tolerance in different populations [4,5,6]. Moreover, the levels of blood gases (i.e., CO_2_ and O_2_) and, thus, the blood’s acidity (pH), induce changes in the RR, according to the respiratory compensation mechanism [7]. Other breathing parameters are important as markers of the user’s health status. In detail, the inhalation (TI) and exhalation (TE) times, as well as their ratio (IER—inhalation-to-exhalation time), are strictly correlated to the heart rate variability, as well as physical and psychological status [8,9,10]. Additionally, the flow rate (V) reflects the patient’s pulmonary capability, which can be affected by several diseases of the cardiorespiratory system (e.g., COPD—chronic obtrusive pulmonary disease) [11].

Likewise, technological advances in the field of sensing technologies and methods for breathing monitoring are growing exponentially, and several measurement solutions are currently available, exploiting various transduction mechanisms (e.g., inertial, capacitive, piezoresistive, humidity, gas sensors, etc.) [12,13,14,15]. Indeed, the scientific community and various companies are constantly trying to develop new breathing monitoring systems that are more discreet and nonobstructive than those commonly used (e.g., spirometers and capnometers). For instance, it has recently been demonstrated that micro-electromechanical system (MEMS) inertial sensors worn on the torso can measure inclination changes due to rotational chest-wall movements during respiratory activity, allowing for breathing monitoring [16,17,18]. In addition, MEMS inertial sensors have been widely integrated into wearable systems, given their enhanced performance obtained in recent decades [19,20,21,22,23]. Piezoresistive and capacitive transduction methods are the most used in MEMS inertial sensors [24,25]. Their strength lies in their versatility and noninvasiveness, making them ideal for various applications and representing a reliable and inexpensive way of collecting users’ motion data [24,26,27].

This scientific work reports on the development of an inertia-based chest band to detect breathing-related chest wall movements using a differential inertial approach. In detail, the developed chest band includes two IMUs (inertial measurement units) positioned on the chest and back, allowing for the detection of breathing movements, regardless of body movements. Additionally, the chest band includes a low-power microcontroller section to acquire inertial data from the two IMUs and process them to extract the breath-related differential signal, using the back inertial sensor as a reference. The firmware parameters were systematically optimized to improve the device’s accuracy. A BLE (Bluetooth low-energy) transceiver wirelessly transmits the acquired breathing parameters (i.e., RR, TI, TE, IER, and V) to a mobile application, where data are displayed and summarized. Finally, several test campaigns are conducted to validate the proposed chest band and approach to detect the breathing parameters. Additionally, a performance comparison is provided with another chest band combining a textile-based piezoresistive sensor and a single inertial sensor for detecting the same breathing parameters reported above [28]. In this way, useful insights related to the more performant and promising sensing technology are deduced.

The novelties of the presented work are different, and are described as follows: The presented differential inertial approach ensures a high immunity to the motion artifacts, as witnessed by the experimental results reported in Section 3. Indeed, the algorithm evaluates the relative acceleration signal between the chest and back and applies digital filters for removing the signal components extraneous to breathing. The resulting firmware is computationally light, enabling its use on hardware with reduced memory and computational power. For these reasons, this approach allows for the accurate and real-time measurement of breathing parameters locally on the chest band without a host device performing postprocessing (e.g., PC), unlike similar solutions presented in the literature. Furthermore, the research activity concerns not only the development and optimization of the firmware, but also the design hardware section and the analysis of sensor placement, unlike other similar scientific works, mainly focused on firmware development. This activity has resulted in a compact, lightweight, complete, and ready-to-use wearable system, as detailed in Section 2.1.

The main contributions of the proposed research work are:

- The development of a novel chest band for monitoring breathing parameters based on a differential inertial approach; this includes a low-power microcontroller section that acquires inertial data from both inertial sensors, processes them to extract breathing data, and coordinates the transmission of data to a custom mobile app.
- Firmware development for acquiring and processing inertial data from the two IMUs. In detail, the processing relies on digitally filtering the acquired data to remove the undesired signal components and applying a peak detection algorithm to detect the inhalation and exhalation times, useful for deriving other breathing parameters.
- A comprehensive characterization of the developed inertia-based chest band for determining its performances in terms of the correlation coefficient, mean difference (MD), limits of agreement (LoA), and mean absolute error (MAE), compared to the reference measurements. In detail, correlation and Bland–Altman analyses were performed on the measures gathered by eight users varying in age, gender, and physical constitution. Additionally, a comparison between the performances of the developed chest band with another one based on a piezoresistive strain sensor and an IMU, presented in our previous work, was carried out [28].

The paper’s remainder is arranged as follows: an overview of wearable sensing devices for monitoring cardiorespiratory parameters is presented. Section 2 introduces the inertia-based chest band’s architecture and the firmware managing the device’s operation. Later, the assembly of the inertia-based chest band is introduced. Section 3 reports on the firmware optimization for improving the chest band’s accuracy; then, the chest band’s characterization and testing are reported on to determine its performance. Finally, in Section 4, the results are discussed, with an emphasis on the advantages and disadvantages of the presented wearable device.

### Overview of Wearable Devices for Monitoring Cardiorespiratory Parameters Based on Inertial Sensors

Accurate breathing monitoring is crucial for monitoring a patient’s health status. However, the most used methods, which have been proven reliable, are typically obtrusive and unsuitable for use outside the hospital environment. For this reason, many studies have focused on finding alternative methods and technologies to current golden standards (e.g., spirometry), which may or may not require body contact. In detail, inertial sensors represent an obstructive, cheap, and accurate solution for realizing systems to detect body movements, such as joints, fingers, the head, etc. [20,29,30,31]. Thanks to the high resolution reached by MEMS inertial sensors, they can monitor very tiny movements, such as the chest movements induced by respiration or the heartbeat [32,33,34,35].

Inertia-based breathing sensors show several benefits compared to other methods, overcoming several limitations. Methods such as impedance pneumography (IP) and electrocardiographic-derived respiration (EDR) require applying electrodes on the user’s skin [36]. These contacts can heavily affect the acquired signal’s quality and, thus, the accuracy of the obtained measurements [37]. In particular, contaminants on the leads can influence the system performance; similarly, cable and wire impedance can be a source of errors. In addition, the measurement depends on natural behaviors (i.e., coughing, talking, etc.) and user posture, whereas body motions and lack in contact can induce artifacts in the acquired signal. Furthermore, the EDR method using RSA (respiratory sinus arrhythmia) is affected by the patient’s age, weakens with advancing age and leading to measurement inaccuracy [38]. Conversely, inertial sensors do not require direct contact with the user’s skin; hence, they are immune to most of the problems described above.

Likewise, photoplethysmographic (PPG) sensors require contact between the sensor’s optical section and the patient’s skin [39,40]; this entails that they suffer from the same problems as EDR and IP approaches related to artifacts induced by body motion and lack of contact. Generally, IMU-based breathing sensors are also sensitive to body motions; however, different strategies are feasible in reducing the effects of motion artifacts, such as the differential approach presented in this paper. Camera-based methods for breathing monitoring surely have the advantage of being contactless, but currently suffer from different issues. In detail, they are inaccurate unless using multiple cameras on different body zones (i.e., thorax, abdomen, lateral side, and back), costly, affected by the patient’s position changes, inefficiency in the case of occlusions (cloths, blankets, etc.), and are complex. Finally, these methods cannot be applied to users engaged in common activities (taking, coughing, etc.) [41].

Several methods have been presented in the scientific literature for measuring respiratory parameters using IMUs. For instance, P. Janik et al. presented a novel wearable sensor for monitoring breathing and the heart rate using a single inertial sensor [42]. The first station included a microcontroller unit (MCU) integrating a BLE radio module (nRF52832) equipped with an LSM9DS1 IMU. The developed system comprised a receiving station based on the NRF52840 MCU, equipped with a MAX30102 pulse oximeter acting as a reference sensor that was also used to measure the SpO_2_. The sensor was placed on the abdominal wall to detect the movement induced by breathing and heartbeat. The processing chain removed the DC (direct current) component, and the resulting signal’s absolute value was calculated. Then, the resulting signal was filtered using a low-pass filter and local peaks were detected, representing the pulse. The experimental tests demonstrated that 0.038 s (i.e., 4% of a single cycle) was the mean difference compared to a cabled transmission. Additionally, S. Beck et al. in [43] presented a novel method to calculate the angle between the quaternions of two IMUs to estimate the breathing rate. By positioning one IMU on the abdomen and the other on the thorax, they reasoned that any motion other than breathing would alter the overall quatern, but not the angle between the two IMUs. On a breakout board attached to an Arduino Board, they employed two InvenSenseTM MPU-6050 three-axis gyroscopes and accelerometers. The relative angle was calculated using the IMUs’ outputs, which were already prefiltered by themselves, and the change in this angle was utilized to determine the respiration rate. The gathered data were transferred to a laptop, where MATLAB was used to apply a tenth-order Butterworth filter on them.

Furthermore, several processing schemes and algorithms were developed for detecting breathing anomalies and disturbances, such as apnea events, involuntary breathing movement (IBM), etc. [44]. In [45], the authors developed a deep learning model based on an advanced machine learning algorithm combined with breathing movements, which is useful for detecting sleep apnea events and estimating their severity. The patch acquires the acceleration data with a 3D accelerometer and microphone with a 60 Hz sampling rate, capturing movements and sounds caused by breathing; it was placed on the suprasternal notch of the subject under test to detect tracheal movements. To estimate the apnea severity, the proposed algorithm extracts morphological features from breathing-related movements to train the deep learning classifier to recognize the various breathing events and, finally, to estimate the apnea hypopnea index (AHI), namely, the number of events per hour. The authors also developed a supervised deep learning classifier for improving its hyperparameters to increase its accuracy. The results were compared with those obtained through the PSG (polysomnography), achieving a 0.86 correlation factor. In [46], the authors developed a technique to classify sleep apnea using a MEMS accelerometer. The dominant component of the acceleration signal, extracted by using PCA (principal component analysis), was analyzed, which required processing on 3 × 3 matrixes.

However, two techniques were developed to lower the computational complexity: the first considers the raw data of the two axes with the most significant variance and applies the PCA to them, reducing the problem to 2 × 2 matrices. The second method involves simply choosing the component with the highest variance.

In [35], E.P. Doheny et al. presented an innovative method to measure the respiratory rate and estimate the position during sleep using an accelerometer placed on the torso. The raw signal from the accelerometer was processed through a 0.5 Hz low-pass filter and a 0.5 Hz notch filter. For the proposed setup, the chest movement could be observed along the *z*-axis, whereas the change in sensor orientation due to breathing was observable by calculating the rotation of the *x*-axis [47]. The maximum MAE on the RR was 2.67 BrPM (breaths per minute) if the sensor was placed on the chest, and 2.25 BrPM if it was placed on the abdomen. Such a system has the defect of not working in the prone position.

In [48], the authors developed a system to calculate the respiratory rate and cough frequency using two sensors consisting of a nine-axis IMU (LSM9DS0) and a MEMS microphone placed on the chest and abdomen. These were processed through a first-order complementary filter at 32 Hz. At first, the average between the abdominal and thoracic displacement angles was computed, obtaining the so-called ventral body cavity angle. To remove the high-frequency components, this was decimated at 5 Hz, and then a 0.1 Hz low-pass filter was applied to remove the baseline wander. The Savitzky–Golay filter was used to improve the signal, applying the algorithm to find the peaks and then calculating the RR. The results were compared with those derived from the PPG, calculating the angle displacement on 3 s windows, and applying a 2 Hz low-pass filter of the twentieth order. A further part of the algorithm was cough recognition: they used an algorithm similar to that used for speech detection. The peaks were searched in the generated waveform, identifying coughs.

Additionally, some solutions were reported in the literature based on multiple inertial sensors for the detection of body movements related to respiratory and cardiac activity [34,49]. In addition, these solutions usually exploit a differential approach, processing the difference in inertial data to remove signal components related to undesired body movements [50,51]. However, this approach involves new issues related to the sensors’ positioning on the body and the integration of sensors into the garment, ensuring nonintrusiveness and accuracy.

In ref. [52], the authors presented a method for calculating the respiratory and heart rate, designed to operate even on athletes during heavy movement. The operating principle involved the application of two sensors, one on the sternum and one on the athlete’s back, perfectly aligned with each other. Therefore, the signal of the rear sensor, induced only from movement, was subtracted from the signal of the front sensor, composed of the component due to movement plus that derived from breathing, thus, resulting in a signal due only to breathing under movement conditions. The front section comprised an STM32F4 microprocessor with integrated DSP (digital signal processing), a BLE transceiver, a flash memory, a voltage regulator, a lithium battery, and a LIS3DSH three-axis accelerometer. Instead, the back device had the same accelerometer and a port for connecting to the front device. The *y*-axis data from the sensor, corresponding to the normal direction of the athlete’s chest, were used, and the difference between the two sensors being to obtain the actual breathing data. They proceeded with peak detection using an embedded algorithm, calculating the breathing rate as the reciprocal of the time elapsed between two peaks, with a 100 Hz sampling frequency. As for the heart rate determination, the sampling frequency was increased to 400 Hz; then, a Butterworth filter with a bandpass between 12 Hz and 28 Hz was applied to the raw signal. Afterwards, this was smoothed using a further Savitzky–Golay filter, thus, obtaining the suitable signal on which to apply the embedded algorithm to detect the peaks, obtaining the heart rate. Compared with the results obtained from a reference spirometer, the tests carried out on athletes engaged in various activities showed that this system was particularly reliable when used during intense sporting activities or very light activities, such as office work. It proved less accurate but still obtained good results for intermediate activities, such as walking, as they are less oxygen-intensive and require less chest movement. The normalized root mean square error (NRMSE) was less than 1.42% between the proposed and reference systems.

Another possible configuration for the RR measurement using a dual acceleration measurement was reported in [53]. In their proposal, the authors recorded the respiratory chest wall movements using a dual accelerometer respiratory monitor. The device consisted of a portable data acquisition board connected to a pair of inertial sensors installed on the user’s sternum. The acquisition sensors consisted of two MEMS capacitive triaxial accelerometers calibrated one at a time with a standard procedure to offset the output and correct any possible systematic errors due to the manufacturing process and physical structure. The sensors were mounted onto two small circuit boards to record the chest wall movements. The accelerometer device was configured to measure the average RR based on the number of respiratory events detected over a 60 s time window. The RR onboard was calculated according to the following procedures: Firstly, the six acceleration signals (X_1_, X_2_, Y_1_, Y_2_, Z_1_, and Z_2_) provided by the two accelerometers were digitized and processed to obtain the difference signal (D). Higher-frequency components were removed through the use of a simple smoothing digital filter (moving average). A low-pass filter with a 2 Hz cut-off frequency was used to smooth the signal and remove any nonrespiratory movements, thus, obtaining the respiratory signal.

Finally, Table 1 reports on a comparison between the scientific works discussed above from the point of view of the number of employed inertial sensors, installation position, main processing blocks, and computational complexity evaluated according to personal evaluations. The aim was to identify the most promising solutions for developing the next generation of wearable devices for monitoring breathing parameters.

According to us, the solutions involving dual-inertia detection [43,52,53] offer several advantages compared to those using a single sensor, especially considering the weaknesses of inertial sensors, namely, the extreme sensitivity to extraneous movements, inducing motion artifacts in the acquired signal. Indeed, the differential solutions allow for the removal of extraneous movements related to body motions, which are common to both sensors. In addition, such scientific works report on very simple processing, mainly involving digital filtering and a time domain analysis [11,19,20]. Our scientific work intends to take a step forward with respect to the existing literature, presenting a fully integrated, low-power, and discreet solution, but, simultaneously, featuring optimal performance for monitoring the user’s breathing activity.

## 2. Materials and Methods

The basic idea was to develop a wearable system that measured breathing parameters using two IMUs placed on the user’s chest and back. The presented chest band was low-cost, small, and accurate, and used a powerful microcontroller for processing the acquired inertial signals to extract respiratory activity. It exploited a dual inertial measurement to determine the slow chest movement regardless of the extraneous body motions. The IMUs worn on the torso were employed to measure the inclination and angular changes during respiration. Indeed, by using two sensors, a differential acceleration measurement could be performed, removing the signal components related to body motion. If a perfect alignment of the coordinate systems of both sensors was achieved, this method would eliminate acceleration measurements introduced by a translational movement that did not belong to respiration. After transforming and filtering the inertial data, the respiratory signal could be extracted; the breathing parameters could be calculated by analyzing the small peaks into the differential inertial signal.

### 2.1. Architecture of the Developed Inertia-Based Chest Band

**Figure 1:**
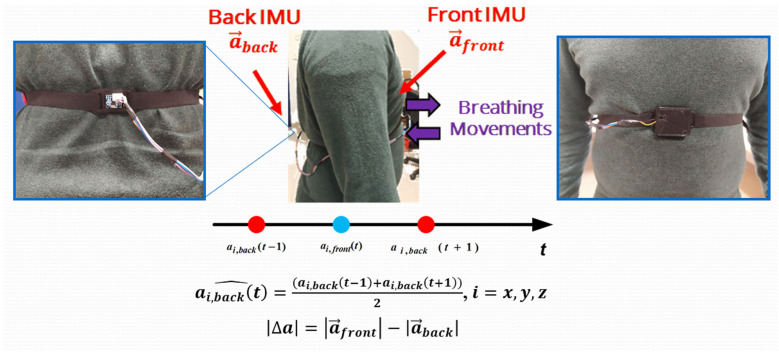
Schematic view representing the operating principle of developed dual-inertia chest band; the red arrows indicate the inertial sensors’ placement, whereas the purple ones the chest movements during the breathing.

The core of the developed wearable device used to measure respiratory frequency was represented by two inertial sensors. Specifically, two six-axis motion tracking devices (model MPU-6050, manufactured by Invensense Co., San Jose, CA, USA) were positioned on the chest and the back of the subject under test and held in position by an elastic band (Figure 1).

The logic behind this device was as simple as it was powerful. The user’s chest expanded and contracted in relation to inhalation and exhalation, featuring the respiration cycle during breathing [54]. Ideally, in conditions of absolute immobility, when fixing an orthogonal reference system of origin on the subject’s chest, the *x*-axis directed along the body, the *y*-axis directed in a normal way with respect to the line of the body with direction outward from the chest, and the breathing movements were along this last axis.

An IMU is perfectly capable of measuring these movements, but when the subject moves, even short movements, the measurement would undergo motion artifacts that were not due to breathing, which could compromise the measurements. To overcome this problem, a second IMU was placed on the subject’s back in exact correspondence with the front sensor. The back was not affected by breathing movements, so all data read by the second accelerometer were exclusively ascribable to the subject’s movements. Therefore, this solution resulted in the first inertial sensor for reading motions and breathing data, with the latter reading only motion data. The raw data on the respiration could be obtained by subtracting the data taken by the front device from the rear one, thus, extracting a respiratory signal regardless of the user’s movements. Afterward, the waveform was initially filtered using the digital signal processing technique to eliminate residual motion artifacts caused by vibrations due to the heartbeat (precordial motion). Then, a peak detection algorithm was applied to the filtered signal to determine the inhalation and exhalation times, thus, allowing for determining the breathing parameters (i.e., RR, TI, TE, IER, and V).

**Figure 2:**
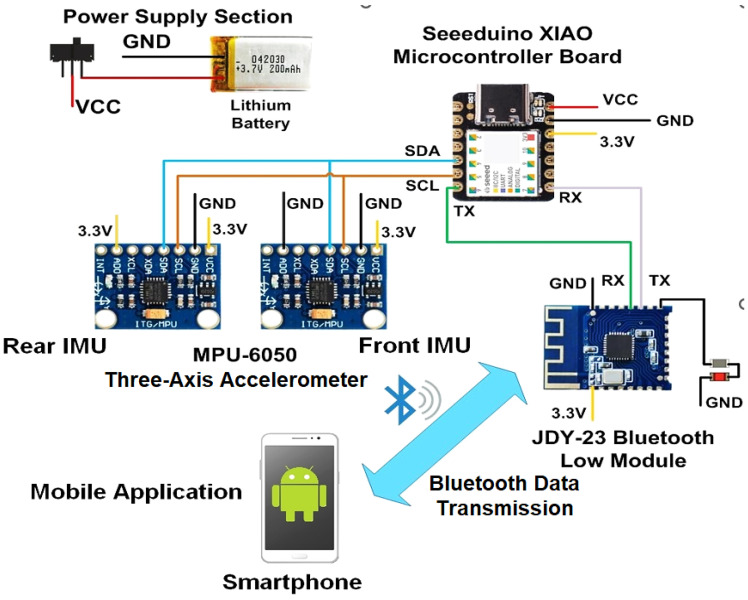
Architecture of the developed inertial-based chest band; the breathing parameters acquired by the chest band are sent through Bluetooth transmission to a mobile application to be viewed.

Detailing the device’s architecture, its core was contained in the front box (Figure 2); a custom-made housing unit contained the electronic acquisition and processing section. This was constituted through a Seeeduino Xiao board, including the SAMD21G18A microcontroller (manufactured by Microchip Inc., Chandler, AR, USA), connected to the first inertial sensor. An MPU-6050 six-axis MEMS inertial measurement unit was employed, combining a three-axis accelerometer, three-axis gyroscope, and a Digital Motion Processor™ (DMP).

Moreover, the microcontroller board was interfaced with a JDY-23 Bluetooth module at 2.4 GHz, and supplied with a single-cell Li-Po battery (model HJ751517, manufactured by Hongjie Electronic Co. Ltd., Kunshan City, Jiangsu, China) featuring a 3.7 V nominal voltage and 100 mAh capacity. On the back, an additional MPU-6050 was connected to the front microcontroller via a cable running along the belt (as shown in Figure 1, image on the left). The belt was essential for holding the two sensors in the correct position. The two inertial sensors were interfaced with the microcontroller through the I^2^C bus, discerned by their 8-bit address.

The microcontroller acquired and processed data from the two inertial sensors and then derived the respiratory wave; it took the acceleration data from the two sensors synchronously, calculated the corresponding inertial differential signal, and filtered it appropriately. It then applied a peak detection algorithm to detect the exhalations and inhalations to calculate the respiratory rate. The integrated Bluetooth module would send the extracted information to a mobile application, allowing the user to display it remotely. A graphic representation of the device architecture is depicted in Figure 2.

The main strengths of the presented inertia-based chest band are listed below:

- Low dimensions and weight: the chest band was compact (6 cm × 2.8 cm × 1.4 cm front section and 4 cm × 2 cm × 0.1 cm rear section) and lightweight (35 g), making it discreet and nonobtrusive.
- Complete and ready-to-use: the development covered both the hardware and firmware sections, leading to a complete monitoring system applicable in real operative scenarios.
- Real-time monitoring: the developed system provided real-time measurements of the breathing parameters without postprocessing through an external host device (e.g., PC).
- High immunity to motion artifacts: thanks to the deployed differential inertial approach, the presented wearable device was relatively immune to artifacts induced by extraneous body movements.

Additionally, the limitations or shortcomings of the presented system concerned:

- The better integration of the rear IMU inside the chest band, improving the wiring, or providing a wireless connection with the front section; in this way, the device wearability would be further enhanced.
- The deployment of advanced digital filtering methods, enabling a better rejection of noise and artifacts induced by body motions.

These considerations are planned to be the starting point for future evolutions of the presented wearable device.

### 2.2. Assembly of the Developed Chest Band

**Figure 3:**
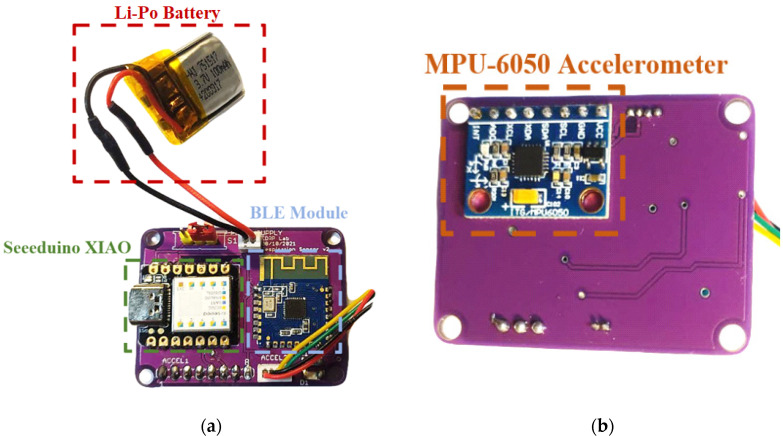
Front (**a**) and backside (**b**) views of the assembled board with main components highlighted.

The PCB (printed circuit board) design of the electronic acquisition and processing section was carried out, placing the Seeeduino Xiao board, the JDY-23 BLE module, and the four-way connector for the rear sensor on the top layer. In contrast, the bottom layer housed the front accelerometer equipping the chest band connected to the SAMD21G18A microcontroller through the I^2^C interface (Figure 3b).

**Figure 4:**
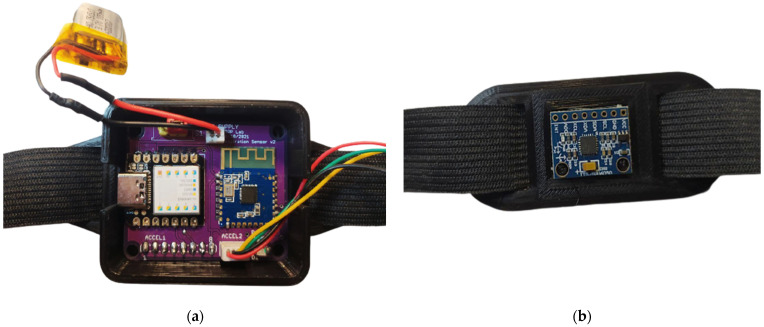
Pictures of the developed chest band: top view (**a**) of the electronic acquisition and processing section; rear (**b**) IMU mounted on proper support.

The PCB was installed in a custom 3D printed case (with size dimensions of 6 cm × 2.8 cm × 1.4 cm) properly designed to be comfortable and wide enough to host the board (Figure 4a). It was equipped with buttonholes to allow the passing of an elastic band used to wear the device. In contrast, the back IMU was installed with proper support and was fixed to the elastic band through two slots (with size dimensions of 4 cm × 2 cm × 0.1 cm) (Figure 4b). Everything was designed to be as comfortable and easy to handle as possible, not to disturb or alter the subject’s breathing or the measurement process.

### 2.3. Firmware of Developed Inertia-Based Chest Band

This section describes the working modalities and firmware development of the proposed wearable device, managed and controlled using a core section represented by the Seeeduino Xiao microcontroller board.

**Figure 5:**
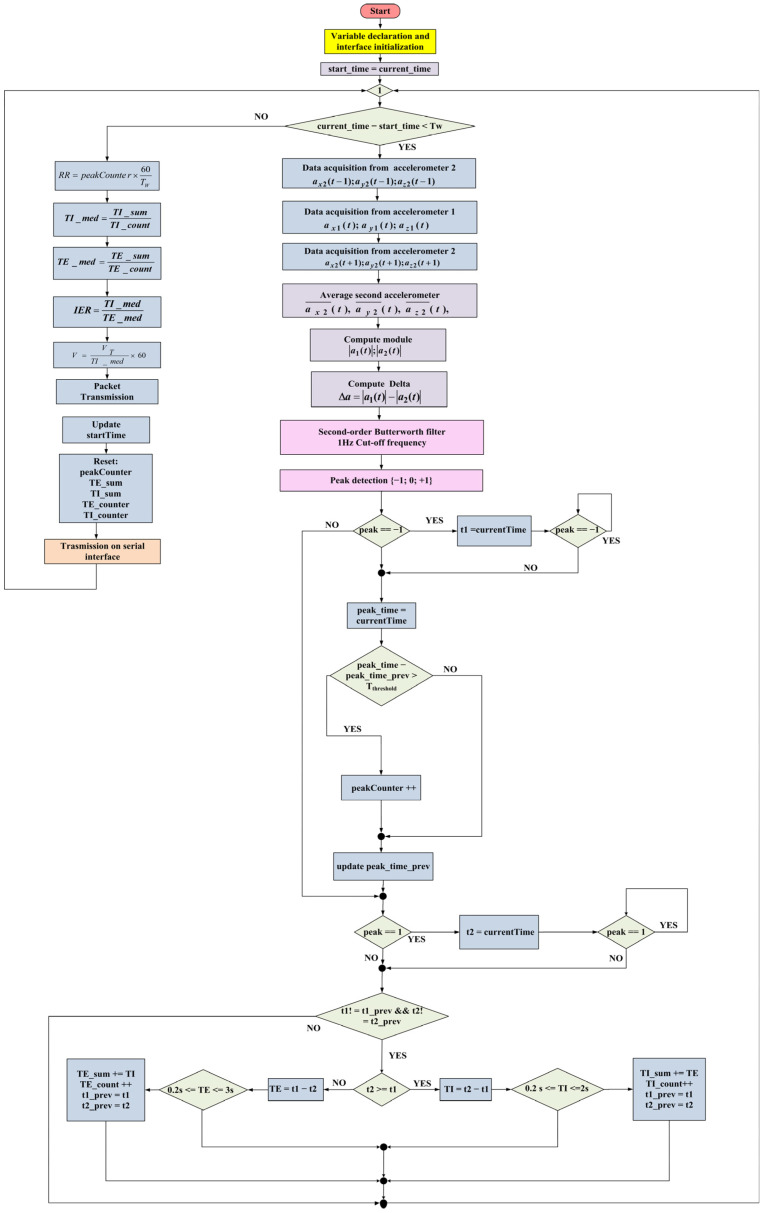
Flowchart of firmware implemented in the inertia-based chest band.

The signals acquired from the accelerometer were just raw data and needed to be processed and analyzed to detect the exhalation and inhalation instants and, thus, the breathing rate. To extract the breathing parameters from the acceleration difference signal, the acquired data had to be filtered to remove extraneous signal components and processed to detect the inhalation and expiration times. Specifically, a digital low-pass filter with a 0.6 Hz cut-off frequency was implemented, since the respiratory signal featured a characteristic frequency between 0.2 Hz and 0.6 Hz. The flowchart of the implemented firmware is shown in Figure 5.

The firmware started declaring variables useful for programming purposes and initializing the communication interfaces. Notably, the I^2^C (interintegrated circuit) and UART (universal asynchronous receiver transmission) interfaces were employed. The first one was used for communicating with the two accelerometers. A supplementary connection between AD0 and 3.3 V was necessary to use the second MPU-6050 to change the default I^2^C address from 0 × 68 to 0 × 69, allowing to discern between the two devices on the I^2^C bus. Moreover, the accelerometer range was configured to be ±2 g, and an integrated 21 Hz low-pass filtering process was activated on the inertial data. The second interface was used to communicate with the BLE module, which allowed for the transmission of the acquired RR measurements to a host device (i.e., smartphone or tablet). Later, a variable called *start_time* was used to store the time when the time window started, and was initialized with the *current_time*. $T_{w}$ is the time window used for the RR estimation, and it was set to 30 s (the value chosen in this thesis); *peak_counter* (or $N^{P}$) is the variable used to count the number of positive peaks occurring in the time window, which was initialized to 0. In contrast, variables *peak_time*, *t1*, *t2*, *t1_prev*, and *t2_prev* were used to store the exhalation time, inhalation time, and the last inhalation and exhalation times, respectively.

Afterward, the program flow entered into a continuous loop to acquire and process the raw data and provide the RR value to the user. Then, a time window ($T_{w}$) started, which first acquired the acceleration data from the two inertial sensors; for obtaining a synchronous acquisition of acceleration data from the two IMUs, two symmetrical measurements were acquired from the rear sensor with respect to the first one. The mean value of the two contiguous measurements from the rear IMU was considered to estimate the acceleration value of the second sensor at the time when the front IMU was interrogated. Specifically, a new data value was acquired from the rear IMU:

(1) $$a_{x,\;\mathrm{back}}\left(t-1\right){;\;a}_{y,\;\mathrm{back}}\left(t-1\right){;\;a}_{z,\;\mathrm{back}}\left(t-1\right)$$

Then, a new data value was acquired from the front IMU:

(2) $$a_{x,\;\mathrm{front}}\left(t\right);\;\;a_{y,\;\mathrm{front}}\left(t\right){;\;a}_{z,\;\mathrm{front}}\left(t\right),$$

Additionally, new data were acquired from the rear IMU:

(3) $$a_{x,\;\mathrm{back}}\left(t+1\right){;\;a}_{y,\;\mathrm{back}}\left(t+1\right){;\;a}_{z,\;\mathrm{back}}\left(t+1\right),$$

This acquisition method reduced the number of errors caused by the nonsynchronous acquisition from the two inertial measurement units. By calculating the average of the acceleration data from the rear IMU, an estimation of the acceleration data detected from the rear IMU at the same time as the front one was obtained (Equation (4)). Therefore, the average of the data from the rear IMU was calculated:

(4) $$\hat{a_{x,\;\mathrm{back}}\left(t\right)};\;\hat{a_{y,\;\mathrm{back}}\left(t\right)};\hat{{\;a}_{z,\;\mathrm{back}}\left(t\right)},$$

Afterward, the modules of the two acceleration vectors were computed:

(5) $$\left|{\vec{a\;}}_{\mathrm{front}}\left(t\right)\right|;\;\left|{\vec{a\;}}_{\mathrm{back}}\left(t\right)\right|,$$

Additionally, the difference between them was as follows:

(6) $$\Delta a=\left|{\vec{a\;}}_{\mathrm{front}}\left(t\right)\right|-\left|{\vec{a\;}}_{\mathrm{back}}\left(t\right)\right|,$$

This acceleration difference signal represents the raw data processed to extract the respiratory parameters. Afterward, the signal was filtered with a real-time digital filter and realized with a Butterworth low-pass filter to remove the frequency components over 1 Hz, thus, removing signal components induced by other body movements. Afterwards, the peak detection algorithm was implemented to detect the inhaling and exhaling phases. The method was based on the deviation principle, detecting if a new data point deviated a given number of standard deviations upward or downward apart from a moving average (also called the z-score). This algorithm output 1 when the average was above the z-score, and 0 if it was within the z-score. When the average was below the z-score, it was marked as −1.

Subsequently, an if condition checked for exhalation peaks (peak = −1), storing the corresponding time (t1); a while loop was introduced to wait for the peak end, avoiding multiple increments. Afterwards, the *peakCounter* was increased if the difference between the current peak time (*peak_time*) and the previous one (*peak_time_prev*) was higher than a given threshold (T_threshold_). This additional verification was intended to eliminate multiple negative peaks that could be seen in the breathing signal due to unrelated body motions. The time threshold indicated above was set to 1.1 s (empirically established value). Then, an if condition checked for inhalation peaks (peak = 1), memorizing the corresponding time (t2). Afterwards, the program flow verified if the inhalation and exhalation times were updated with respect to their previous value. According to the values of the inhalation and exhalation instants, the current inhalation (*TI*) and exhalation (*TE*) time durations were calculated. Additionally, in this case, conditions were applied to the TI and TE values with thresholds dictated through physiological reasons. If the obtained TI and TE values were acceptable, they were accumulated in storage variables (*TI_sum*, *TE_sum*) and used to calculate the mean values on the time windows; then, the *t1_prev* and *t2_prev* variables were updated.

Once the time window ended, the breathing parameters were calculated using the following equations:

(7) $$\mathrm{RR}=\mathrm{peakCounter}\;\times \;\frac{60}{T_{w}}$$

(8) $${\mathrm{TI}}_{\mathrm{med}}=\frac{{\mathrm{TI}}_{\mathrm{sum}}}{{\mathrm{TI}}_{\mathrm{count}}},$$

(9) $${\mathrm{TE}}_{\mathrm{med}}=\frac{{\mathrm{TE}}_{\mathrm{sum}}}{{\mathrm{TE}}_{\mathrm{count}}},$$

(10) $$\mathrm{IER}=\;\frac{{\mathrm{TI}}_{\mathrm{med}}}{{\mathrm{TE}}_{\mathrm{med}}},$$

(11) $$V=\;\frac{V_{t}}{{\mathrm{TI}}_{\mathrm{med}}}\times 60,$$

As evident, the RR was calculated using Equation (7) by counting the number of inspirations that occurred inside the time window (i.e., 30 s); hence, it had to be considered a mean value over the observation interval.

The user’s height and the ideal body weight (IBW) were used to determine the tidal volume ($V_{t}$) [55]. Equation (14) used the IBW to compute the tidal volume:

(12) $$\mathrm{IBW}=50\;\mathrm{Kg}+0.91\times \left(h\left[\mathrm{cm}\right]-152.4\;\mathrm{cm}\right)\;\;\;\;\;(\mathrm{for}\;\mathrm{men}),$$

(13) $$\mathrm{IBW}=45.5\;\mathrm{Kg}+0.91\times \left(h\left[\mathrm{cm}\right]-152.4\;\mathrm{cm}\right)\;\;\;\;(\mathrm{for}\;\mathrm{women}),$$

(14) $$V_{t}=7\;\mathrm{mL}/\mathrm{Kg}\;\times \;\mathrm{IBW},$$

The ratio of the air volume carried during each respiratory cycle ($V_{t}$, expressed in liters) to the inhalation time (TI, expressed in minutes) determined the flow rate (V, in LPM liter per minute). Finally, the data were transmitted to the host device through the BLE transceiver. Then, all variables were cleared and the *start_time* was updated to restart a new time window. Due to the resultant firmware being computationally light, it could be used on hardware with less memory and processing power. Indeed, it covered approximately 53 KB of program memory (19% of SAMD21G18A flash memory) and 16 KB of data memory (50% of SAMD21G18A SRAM (static random access memory)).

## 3. Results

**Figure 6:**
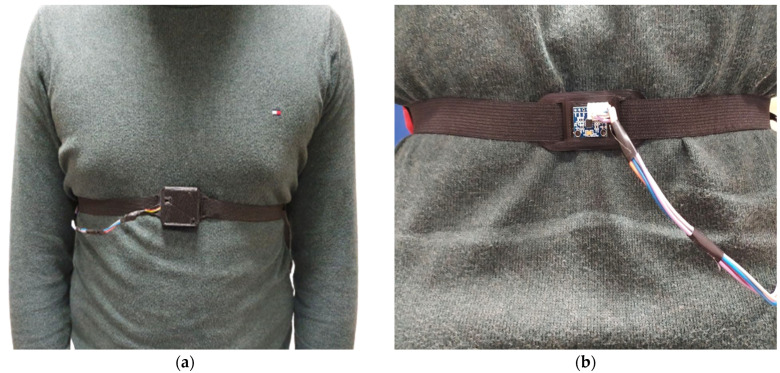
Front (**a**) and back (**b**) view of the inertia-based chest band for measuring respiratory rate.

Section 3 describes the evaluation and testing of the hardware and firmware of the presented dual-inertia chest band. In detail, eight healthy patients different in gender (four male and four female), body constitution (72.1 ± 12.0 Kg), and age (24.3 ± 1.5 years) were considered for the device characterization and testing, as well as for the firmware optimization (Table 2). Depending on the user’s physical structure, the band was worn on the upper abdomen of each wearer, with the elastic band being adjusted to guarantee the proper fit of the inertial sensors on the body (Figure 6). The firmware performed a preliminary assessment as soon as the chest band was placed on to ensure that it was properly positioned on the user’s body when the patient was standing or sitting. The algorithm specifically checked the differential inertial signal produced by the modulus difference of the triaxial acceleration data, which was proximal to zero, indicating a good alignment between the front and rear IMUs. The chest band could be worn by people of varied constitutions and genders thanks to this control system, which maintained the device’s functionality. For all the considered users, a correct calibration was obtained. In addition, a portable spirometer (model SP10, manufactured by Contec Medical Systems Co., Ltd., Qinhuangdao, China) was employed as a reference for the measurements acquired with the chest band collected simultaneously.

### 3.1. Firmware Optimization for Enhancing the Chest Band’s Accuracy

Based on several tests carried out in the laboratory, the chest band’s firmware was improved by optimizing the involved parameters. To improve the algorithm accuracy, and, consequently, that of the device, in detecting the respiratory frequency, it was decided to work on five specific parameters included in the algorithm. The combination of these had an important effect on the accuracy of the same: the cut-off frequency, the peak detector’s parameters, and the minimum distance between two consecutive peaks.

The first parameter was the cut-off frequency of the low-pass filter, used to exclude high-frequency components. Most scientific papers dealing with this subject using a respiratory frequency ranging from 0.5 Hz to 0.8 Hz demonstrated this to be the most appropriate to describe respiratory behavior, excluding motion artifacts. The algorithm discussed in this work was developed by setting the cut-off frequency to 0.5 Hz.

The parameters set in the peak detection algorithm had the greatest effect on the algorithm’s accuracy. This function admitted three main parameters; lag, threshold, and influence; the meaning of which was explained in the previous section. The starting parameter set was 56 samples for the lag, 2.95 σ for the threshold, and 0.58 for the influence.

Additionally, the other considered parameter was the minimum distance between two adjacent peaks ($T_{\mathrm{threshold}}\;$in Figure 5). This time distance should have been at least 1100 ms; experimentally, it was noted that, occasionally, a single inhalation/exhalation generated two peaks in the respiratory waveform, which were erroneously considered by the firmware to be associated with two breaths. Therefore, it was decided to remedy this false detection at a firmware level by adding a condition that discarded the second peak if it was less than 1100 ms away from the previous one. This time interval was crucial, since a too small a value could cause the double counting of the same breathing event, and, in contrast, a too-high value could erroneously discard valid peaks, especially during faster breathing, such as that during the expenditure of physical effort.

Initially, tests were conducted using the starting parameter set with the subject sitting, standing, and moving, obtaining a correlation coefficient between reference and band measurements of 0.859, 0.897, and 0.864, respectively. Therefore, the device demonstrated accuracy and reliability, although the results left room for improvement.

With a view towards improvement, the parameters listed above were adjusted to correct any under or overestimation of the sensors. On-field tests were carried out for each set of parameters. The tests were carried out by simultaneously measuring, over a 30 s time window, the RR with the developed chest band and a portable spirometer (model SP10, manufactured by Contec Medical Systems Co., Ltd., Qinhuangdao, China) used as a reference. Table 3 shows the different parameter sets tested. Indeed, Table 4 reports a comparison between the RR measurement provided by the chest band and the reference ones for each parameter set. These measurements were gathered for a single subject (user one, Table 2) considered in our analysis.

As shown in Table 4, the fifth parameter gave the best results, proving the closest possible results compared to manual measurements. Therefore, it was decided to investigate the results obtained by further testing this parameter set.

### 3.2. Characterization of the Developed Inertia-Based Chest Band

After optimizing the parameters involved in the chest band’s firmware, the next step was to evaluate the performance of the inertia-based chest band in detecting breathing parameters for different user conditions, namely, when sitting, standing, and walking. These tests aimed to assess the correct operation of the developed hardware and firmware when the system was subjected to mechanical stresses or placed in different orientations.

**Figure 7:**
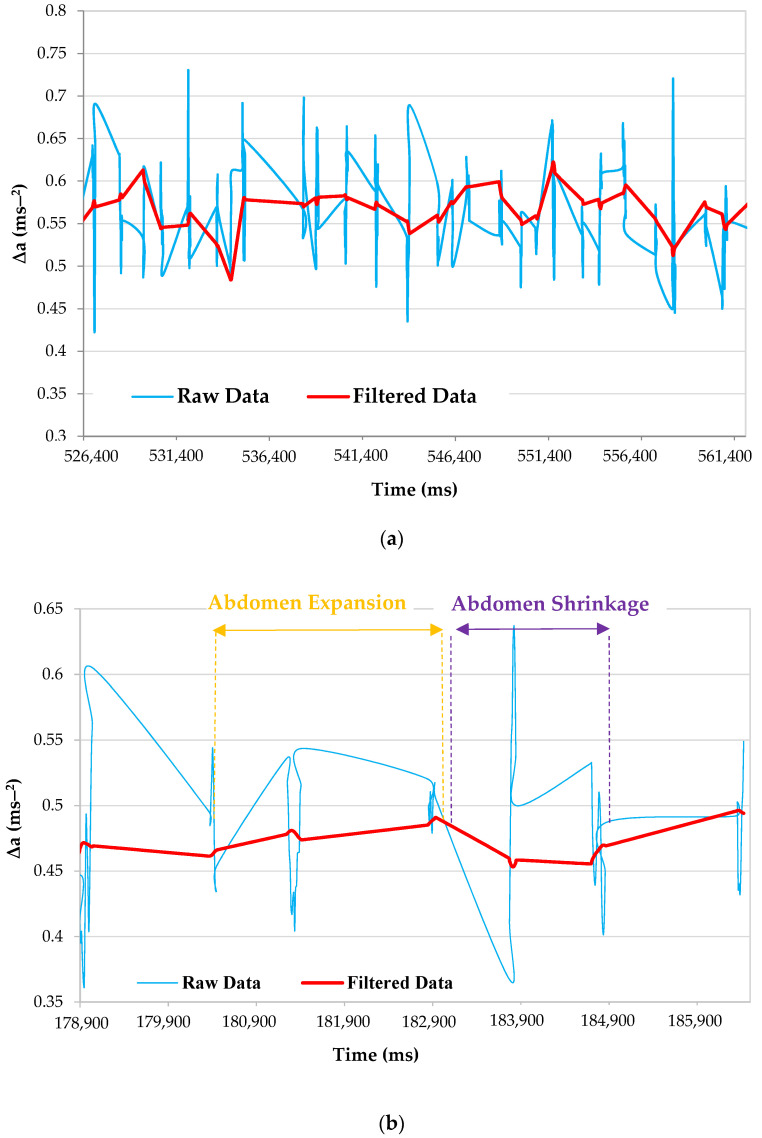
Plots showing the raw data (blue trace) and filtered data (red trace): wide (**a**) and detailed (**b**) views, with the inspiration and expiration phases highlighted.

In Figure 7a, the raw inertial signal, namely, the Δa, is shown (blue trace); the latter, given by the difference between the data obtained from the front and rear sensors, contained high-frequency components, partly due to the heartbeat and uncompensated body movements. As mentioned, these unwanted components were removed through a Butterworth filter at a frequency of 0.6 Hz. The filter action was evident in Figure 7a (red trace), representing the filtered signal deprived of the disturbing components with a frequency higher than the cut-off frequency.

The front inertial sensor experienced a positive acceleration along the *z*-axis as the abdomen expanded due to breath inspiration, increasing the acceleration modulus difference signal. In contrast, as the abdomen contracted during expiration, the front IMU experienced a negative acceleration along the *z*-axis, because it tended to return to its initial position, thus, reducing the resulting differential signal (Figure 7a). Figure 7b provides a more in-depth view of the collected waveforms.

**Figure 8:**
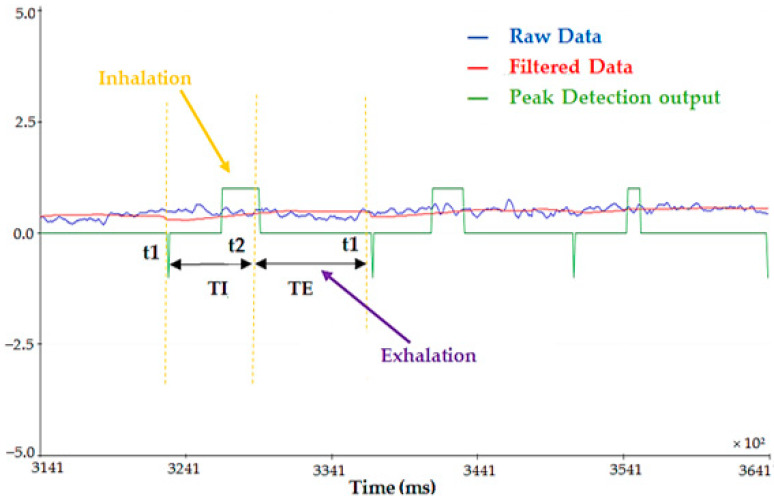
Plot depicting the raw (blue trace) and filtered (red trace) acceleration signals, along with the output of the peak detection algorithm (green trace) based on the dispersion principle.

Additionally, because the peak detection technique was based on a statistical data analysis, by detecting the points’ deviations from the moving average, the processing chain was comparatively insensitive to tiny signal amplitude decreases, ensuring proper device performance. Specifically, it showed the exhale and inhalation instants used by the firmware to generate the respiratory parameters. The green trace represents the output signal from the peak detecting algorithm operating according to the dispersion principle (Figure 8), providing a value of −1 in correspondence to exhalation and +1 in correspondence to inhalation. Observing both the red (filtered ∆a) and green traces simultaneously, it could be deduced that the peak detector picked up the exhalation peak at the point where the green trace stopped growing, and decreased almost instantaneously. The inspiratory peak was much longer, given that an inhalation phase lasted much longer than an exhalation one. In addition, specific time instants of the respiratory signal and the related time length were highlighted in Figure 8. As detailed above, the method created used these data to determine the instantaneous TE and TI, as well as the corresponding average values, the IER, and the flow rate in a 30 s time window.

**Figure 9:**
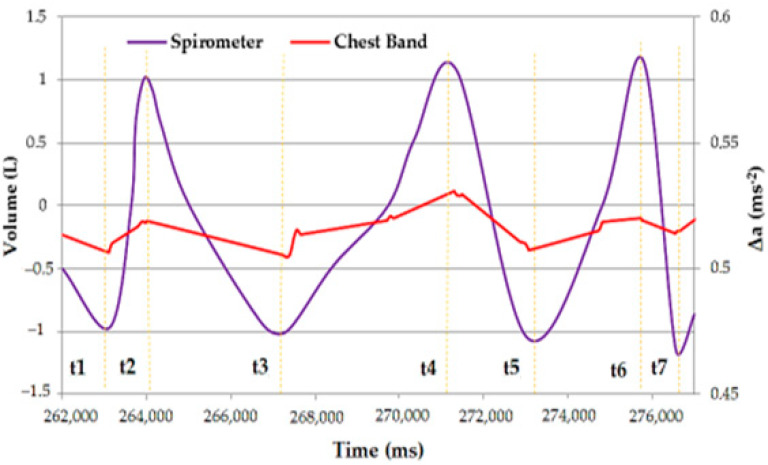
Comparison between the time trend inhaled/exhaled air volume and differential acceleration signal acquired using the chest band; the peaks in inhaled/exhaled air volume are highlighted.

Moreover, the signal produced by the chest band was compared with the time trend of the inhaled and exhaled air volume concurrently acquired with a portable spirometer (Figure 9). As evidenced, the two signals showed similar patterns, exhibiting peaks and troughs at very close temporal positions; thus, we could conclude that the developed wearable device correctly recognized the breathing motions.

**Figure 10:**
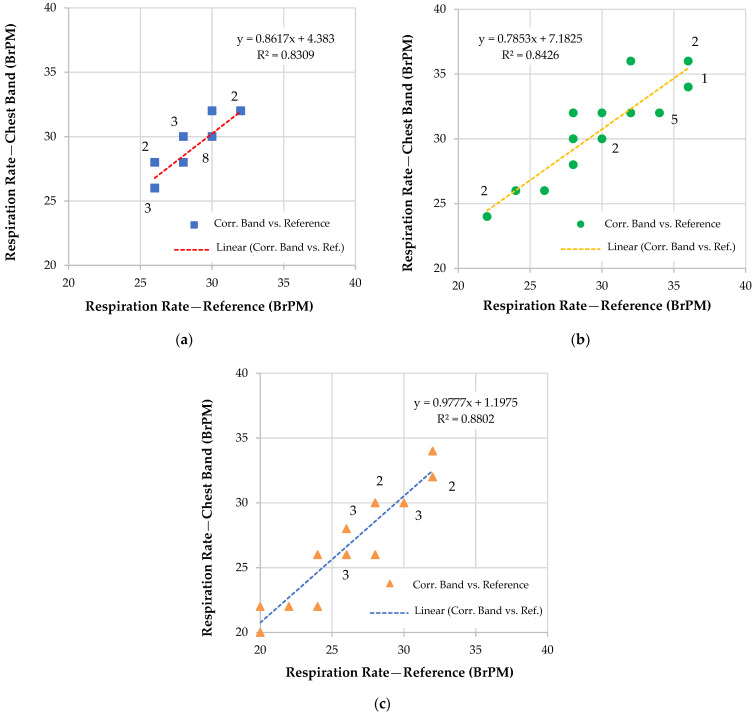
Correlation diagrams between measurements obtained from the chest band and the reference ones obtained with the spirometer for three operative scenarios, namely, for a user: seated (**a**), standing (**b**), and walking at 1 ms^−1^ (**c**). The data point’s multiplicity is reported in the graphs.

Then, the designed inertia-based chest band was tested in various operating scenarios, including sitting, standing, and walking. Twenty tests were conducted for each operating condition, comparing the RR readings produced by the chest band with those acquired by the spirometer. Figure 10 shows the correlation diagram of the measurements provided by the developed wearable device with the reference one. In these tests, only user one (Table 1), an adult man of 24 years and weighing 78 Kg, was involved. Later, a summarizing table is presented reporting on the performance of the chest band for the eight tested participants. To cover a wider RR range, the tests were repeated in various physiological situations (rest and stress); in the graphs, the number of each data point was shown.

Moreover, the correlation between the acquired RR values with the reference ones was quantified through the Pearson correlation coefficient, expressed as (Equation (15)):

(15) $$r=\frac{n(\sum {\mathrm{RR}}_{\mathrm{Band}}{\mathrm{RR}}_{\mathrm{Ref}})-(\sum {\mathrm{RR}}_{\mathrm{Band}})(\sum {\mathrm{RR}}_{\mathrm{Ref}})}{\sqrt{\left[n\sum {\mathrm{RR}}^{2}{}_{\mathrm{Band}}-{\left(\sum {\mathrm{RR}}_{\mathrm{Band}}\right)}^{2}\right]\left[n\sum {\mathrm{RR}}^{2}{}_{\mathrm{Ref}}-{\left(\sum {\mathrm{RR}}_{\mathrm{Ref}}\right)}^{2}\right]}}$$

**Figure 11:**
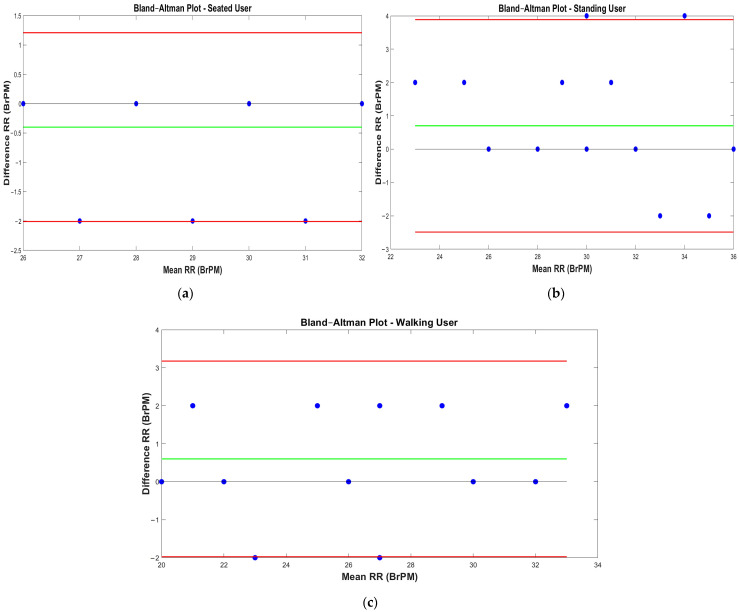
Bland–Altman plots of the RR measurements obtained using the developed chest band and the reference ones for different operating conditions: seated user (**a**), standing user (**b**), and a 1 ms^−1^ walking user (**c**); the blue dots represent the data points, whereas the green and red lines the MD and LoA values, respectively.

Additionally, Figure 11 shows the Bland–Altman plots related to the RR measurements acquired through the use of the inertia-based chest band and the reference ones for different operative conditions (i.e., seated, standing, and walking). These data representations enables the evaluation of the accordance between the two considered measurement methods through two parameters, namely, the LoA and MD.

**Figure 12:**
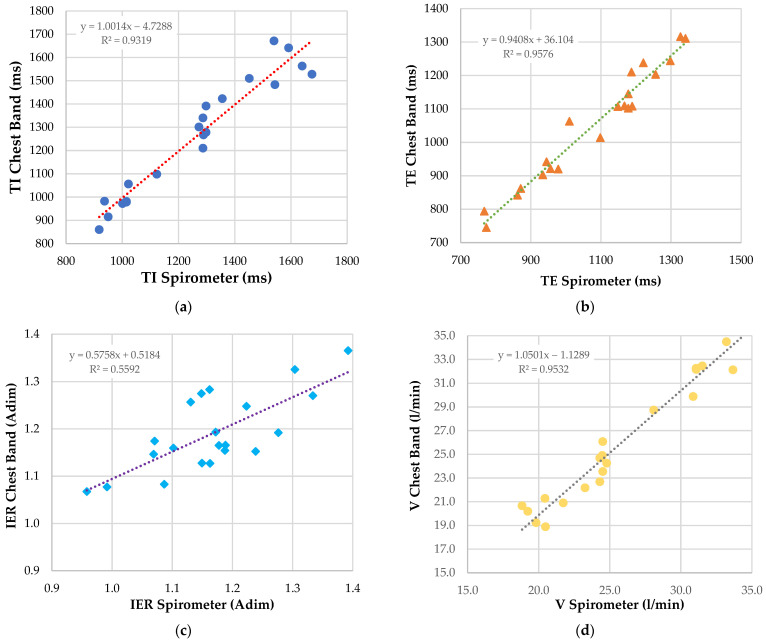
Correlation diagrams related to the measurements of TI (**a**), TE (**b**), IER (**c**), and V (**d**), carried out through the use of the developed chest band and the spirometer used as reference. The colored symbols (i.e., blue and yellow dots, orange triangles, and blue squares) indicate the data points.

Later, the performances of the developed chest band in monitoring the other breathing parameters (i.e., TI, TE, IER, and V) were evaluated. The data provided using the portable spirometer constituted the reference, and were acquired simultaneously with those measured from the presented chest band. Similarly, the measurements were gathered on a single patient (user one in Table 2); correlation analyses were performed to evaluate the chest band performances over 20 tests (Figure 12). In particular, the TI, TE, and V measurements were taken over a single breath.

## 4. Discussion

This section discusses the experimental results of firmware optimization and device characterization presented in Section 3. Furthermore, a performance comparison with the experimental results was conducted in our previous work [28] regarding a piezoresistive and inertial chest band for monitoring the same breathing parameters as that presented in this scientific work. Finally, the performance of the chest band on heterogeneous patients was evaluated.

The following observations could be deduced considering the correlation diagrams of Figure 10: When sitting, the correlation coefficient obtained was 0.911552 (0.859 for set 0, +5.7%), whereas, when standing, it was 0.917919 (0.897 for set 0, +2.3%), and when walking, it was 0.938202 (0.864 for set 0, +7.9%). Therefore, it could be concluded that there was a tangible improvement in the device’s performance using parameter set 5 compared to the previous set 0. Additionally, the device’s tendency to be more accurate when the user was standing or walking rather than sitting was confirmed. In conclusion, it could be stated that the device’s accuracy improved considerably through the optimization of the five parameters described above, obtaining an accurate and reliable RR measurement.

However, the measurement disagreement was ascribable to the spurious peaks created by body motions, which were not removed in the inertial difference signal. Indeed, the main problem of this approach to track the user’s respiration laid in the correct positioning of the chest band; in fact, a perfect alignment between the two inertial sensors was the key to achieving the optimal operation of the device. In addition, disturbing factors were represented by a user’s incorrect posture or accidental band movement.

Furthermore, considering the Bland–Altman plots reported in Figure 11, it was possible to draw conclusions about the data concordance between the chest band and the reference measures [56]. In detail, the results suggested that when the user was seated, the device resulted in a −0.4 BrPM mean difference and +1.21 and −2.01 BrPM limits of agreement (Figure 11a). Conversely, when the user was standing, an MD of +0.7 BrPM and LoA of +3.86 BrPM and −2.49 BrPM were obtained (Figure 11b). Thus, the results indicated that when the user was standing, the chest band performance degraded; this was testified through the greater moduli MD and LoA, indicating that the resulting RR measurements were affected by a larger bias component and a larger data dispersion around the MD. In conclusion, for a walking user, an MD of +0.6 BrPM and LoA of +3.18 and −1.98 BrPM were obtained (Figure 11c). The results suggested that the developed device had a lower bias when the user was seated than in an upright position. In addition, the greater LoA reflected a greater potential inaccuracy in the RR measurement. This performance degradation was ascribable to a misalignment in the front and rear IMUs due to the band’s incorrect positioning or the user’s posture.

When comparing the chest band’s performance when the user was standing and walking, a slight performance improvement was obtained when the user was moving, attributable to the differential cancellation mechanism allowed by the deployed differential inertial solution. By observing the Bland–Altman plots for all the operating conditions, the measurement points appeared to be equally distributed when the mean value changed, suggesting that the error between the two measurement approaches was uniform as a function of the measurement value. Finally, the results showed that the presented wearable device was trustworthy, accurately estimating the breathing rate under situations encountered in daily life.

Moreover, by analyzing the correlation diagrams in Figure 12, the Pearson correlation coefficient could be calculated for the other breathing parameters measured with the chest band (Table 5). As evidenced, the developed device correctly measured the TI, TE, IER, and V, as indicated by the high value of the correlation coefficient of measurements acquired with the chest band and the reference ones. A fortiori, this was evidenced by the low value of the MAE contained within 5% for all considered parameters.

### 4.1. Characterization of the Chest Band for Multiple Users

Later, the wearable device was evaluated over a wider range of users, with differences in gender, physical constitution, and age (Table 2). The aim was to bring out the eventual dependencies of the device’s behavior on user characteristics.

**Figure 13:**
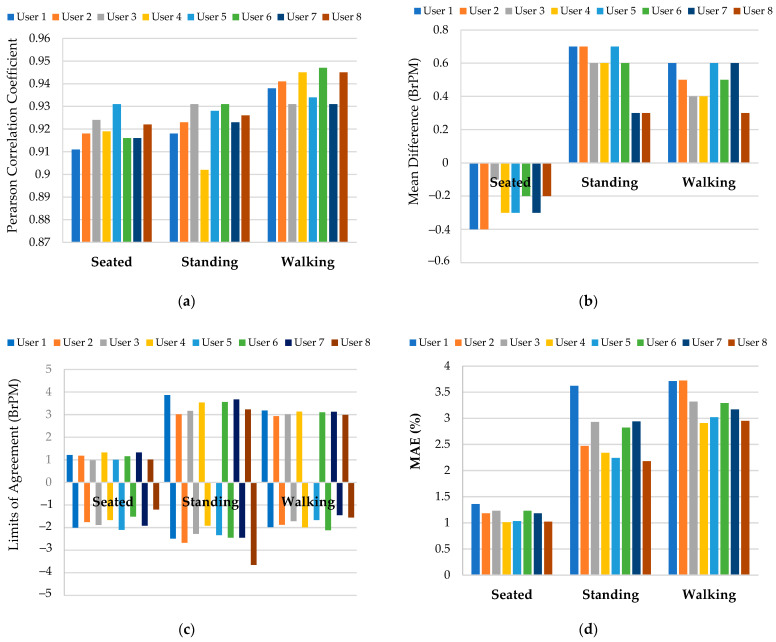
Histograms reporting on the results of the chest band characterization over eight users under different operating conditions (i.e., seated, standing, and walking); Pearson correlation coefficient (**a**), mean difference (**b**), limits of agreement (**c**), and percentage mean absolute error (**d**).

The RR was considered a key parameter to determine the device’s performance. The operational testing modalities were the same as those used for the earlier subject (user one); similarly, the RR measurements acquired with the spirometer were taken as a reference. In terms of the MD, LoA, and MAE, Figure 13 shows the results of the chest band’s characterization obtained for eight users under the previously considered operating conditions (i.e., seated, standing, and walking).

Figure 13 shows that no perceptible correlations from the user characteristics (gender, constitution, and age) were evident in the performance metrics. Moreover, the trends obtained for r, MD, LoA, and MAE were in line with those obtained for user one for the different operative conditions. In detail, the results suggested that the chest band had the best performance when the user was seated, as witnessed by the lowest LoA and MAE. This observation could be justified with fewer mechanical solicitations to which the developed wearable device would undergo, as well as the better posture assumed by the user, which affected the device’s accuracy. Finally, when the user was seated, the mean performances obtained by the inertia-based chest band were as follows: 0.92 mean Pearson correlation coefficient ($\bar{r}$), −0.27 BrPM mean MD ($\bar{\mathrm{MD}}$), 1.16 and −1.75 BrPM mean LoA ($\bar{\mathrm{LoA}}$), and 1.5% mean MAE ($\bar{\mathrm{MAE}}$).

**Figure 14:**
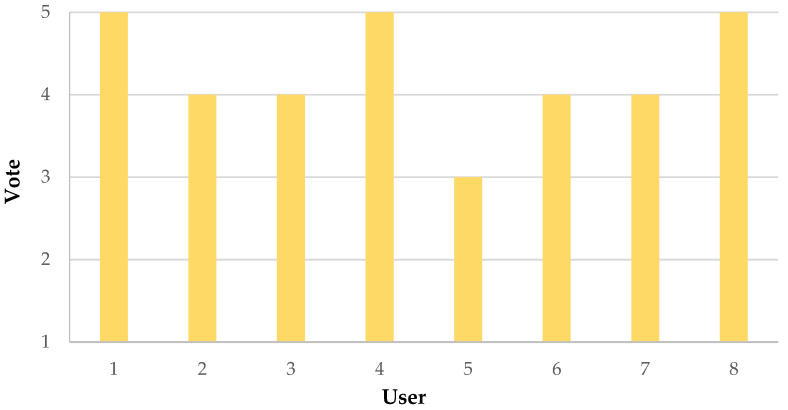
Histograms depicting the survey results related to the comfort of the developed differential inertial chest band.

Finally, we asked the eight considered users to evaluate the comfort of the developed chest band, assigning a vote from one to five; in detail, the meaning of the scale was the following: one (completely uncomfortable), two (more uncomfortable than comfortable), three (more comfortable than uncomfortable), four (moderately comfortable), and five (completely comfortable). The results of the survey are summarized in Figure 14. Each user wore the chest band continuously for one hour, placing it under the clothes; after this period, the user was asked to rate the device’s comfort.

As evidenced from the results in Figure 14, all the users rated the comfort of the developed chest band positively, obtaining a mean score of 4.25. However, much room for improvement of the device’s comfort was deemed feasible, mainly related to the wiring of the rear IMU with the front section, or substituting it with a wireless connection, as discussed in Section 2.1.

### 4.2. Performance Comparison of the Presented Chest Band with the Scientific Literature

This section presents a comparative analysis of the designed dual-inertia chest band with our piezoresistive/inertial breathing sensor [23] to determine the differences and potentialities of the two detection methods. Then, the performance comparison of our inertia-based breathing sensor with similar solutions reported in the scientific literature is introduced to evaluate its capabilities, features, and future perspectives.

The chest band employed a single piezoresistive strain sensor for patient breath monitoring applications based on the EeonTex smart textile (model LTT-SLPA-20K, created by Eeonyx Inc., Pinole, CA, USA) and low-power conditioning, processing, and communication sections. Furthermore, the device integrated an IMU (MPU-6050) to remove motion artifacts, improving its accuracy and reliability in detecting breathing parameters (i.e., RR, TI, TE, IER, and V). Indeed, every motion of the patient’s body unrelated to breathing could introduce components into the collected signal and lead to erroneous measurements of the target parameters. Even when the user was seated or lying down, body motions brought on by the trunk’s bending or twisting could impact the strain sensor’s signal. Notably, the microcontroller section elaborated on the data from the piezoresistive strain sensor jointly with the inertial data for measuring the breathing parameters [28,34].

The performances of the two chest bands, derived from the correlation and Bland–Altman analyses, are reported in Table 6 to examine them and reveal opportunities and potential futures. Specifically, the comparison was carried out considering the RR as a key parameter and evaluating the $\bar{r}$, $\bar{\mathrm{MD}}$, $\bar{\mathrm{LoA}}$, and $\bar{\mathrm{MAE}}$ for three testing conditions, as previously discussed.

It was clear from the results reported in Table 6 that the differential inertial chest band provided improved performance compared to the piezoresistive/inertial chest band when the user was seated. This observation was justified by the lower $\bar{\mathrm{MAE}}$ (−63.2%), $\bar{\mathrm{MD}}$ (−60.3%), and LoA (−63.7% and 0%, respectively). These results indicated that the RR measurements were affected by a lower bias and possible errors determined by the sensing device. In contrast, for the standing user, the performances of the differential inertial chest band were still better than the piezoresistive/inertial one, although to a lesser extent than in the case of the seated user. Indeed, the $\bar{\mathrm{MAE}}$ that resulted from the differential inertial chest band was lower than the piezoresistive/inertial one (−24.4%), whereas the values of $\bar{r}$ were (+3.5%), $\bar{\mathrm{MD}}$ (+28.6%), and LoA (+4.2% and −1.2%, respectively). Finally, considering the walking user, the differential inertial chest band also offered slightly better performance compared to the piezoresistive/inertial one in this case, witnessed by the lower $\bar{\mathrm{MAE}}$ (−8.4%) and LoA (−17.3% and −45.12%), keeping close values of $\bar{r}$ (+1.1%). Nevertheless, considering the walking users, a larger $\bar{\mathrm{MD}}$ (+108.7%) affected the RR measurements as a bias component.

In conclusion, we could state that the differential inertial chest band guaranteed an overall better performance with respect to the piezoresistive/inertial device presented in our previous work [28]. Moreover, the presented detection approach offered several advantages compared to relying just on strain sensors, such as, for instance, the need for no tight coupling with the user’s body, enabling an easier integration inside garments.

Later, a comparison was conducted with similar devices for detecting breathing parameters based on inertial sensors, shown in Table 7; specifically, the percentage of $\bar{\mathrm{MAE}}$ was chosen as a yardstick between the different devices considered, together with the number of used inertial sensors and their positioning, the processing unit, the acquisition frequency, and the availability of a wireless connectivity module. We believed the percentage of the MAE to be the most suitable quantity to evaluate the accuracy of the RR detection devices, allowing to take into account the extent of the committed error commensurate with the respiratory rate measurement.

The presented device is a ready-to-use system for detecting breathing parameters, unlike other solutions presented in the literature, which focused on the detection method, leaving out the applications of the wearable device. In addition, the main advantage of the presented breathing sensor was its real-time processing capability compared to similar solutions, which relied on the postprocessing of acquired data through algorithms performed on a PC [35,43,57,58,59,60]. On the contrary, our chest band processed the data in real-time, providing the mean breathing parameters (i.e., RR, TI, TE, IER, and V) at the end of the 30 s. Furthermore, the presented inertial chest band offered better performance than most of the considered systems (Table 7); only the system presented in [43] guaranteed a slightly better performance (−22.6% on $\bar{\mathrm{MAE}}$) compared to our wearable device. Still, the differential inertial approach in [43] was based on the offline postprocessing that was carried out through a MATLAB script on acceleration data acquired from the inertial sensors.

Furthermore, the presented chest band was equipped with a low-power BLE module for wirelessly transmitting the measurements to a host device. A large part of the reported scientific works did not have wireless connectivity, but the data transfer was entrusted to an SD card or on a PC via cable [43,57,58,60]. In addition, other works employed commercial wearable sensors for gathering the RR measurements, which, however, showed a lower performance than our system [59,61]. Furthermore, the device in [60] was applied to the neck, which could easily be subjected to mechanical stresses that could induce artifacts on the acquired inertial signal. Finally, in [61], the authors employed smart glasses equipped with an IMU to extract RR measurements, which were more obtrusive than our chest band.

## 5. Conclusions

Accurate breathing monitoring is important to determine the onset of critical physical conditions. In the scientific literature, several wearable devices for breathing monitoring have been presented, operating according to different transduction methods involving contact (e.g., oximetry, ECG, inertial, capacitive, piezoresistive sensors, etc.) or not (humidity and gas sensors, video-assisted methods, etc.) with the user’s body.

This paper presented a novel inertia-based chest band for monitoring breathing parameters (RR, TI, TE, IER, and V). Notably, the device employed a differential inertial approach, placing two IMUs on the chest to detect the chest movements and back, acting as a reference. In fact, this approach was based on analyzing the relative acceleration signal between the back and the chest to eliminate signal components unrelated to breathing, enabling a high immunity to artifacts induced by human motions. The chest band comprised a microcontroller section, which acquired and processed the differential acceleration modulus for extracting the breathing parameters. The developed firmware relied on digital filtering and the time domain analysis of the inertial signal; its parameters were optimized to improve the device’s accuracy and reliability. In conjunction with the firmware development, the hardware section was developed, resulting in a compact, lightweight, discreet, and ready-to-use wearable breathing sensor. The chest band was tested on eight users of different genders, ages, and physical constitutions. Correlation and Bland–Altman analyses were employed to determine the device’s performance, using a portable spirometer as a reference. The experimental tests demonstrated that, for seated users, the differential inertia-based chest band could reach a high correlation ($\bar{r}\;$= 0.92) and reduced the mean difference ($\bar{\mathrm{MD}}\;$= −0.27 BrPM), limits of agreement ($\bar{\mathrm{LoA}}\;$= +1.16/−1.75 BrPM), and mean absolute error ($\bar{\mathrm{MAE}}\;$= 1.15 BrPM) in measuring the RR. Additionally, the developed system was compared with another piezoresistive/inertial chest band presented in our previous work [28], showing better performances (−63.2% $\bar{\mathrm{MAE}}$, −60.3% $\bar{\mathrm{MD}}$, and −63.7% and 0% $\bar{\mathrm{LoA}}$). Moreover, the experimental results indicated that the developed chest band correctly detected the other breathing parameters (TI, TE, IER, and V), ensuring an MAE of ≤5%. Finally, we could conclude that the developed differential inertial chest band represented an optimal solution for long-term breathing monitoring in stationary and moving users.

As future developments, the band’s firmware could run machine learning algorithms to discern the occurrence of respiratory disorders (e.g., apnea, snoring, asthma, COPD, etc.) [62,63]. Indeed, thanks to the many available software tools, it is relatively simple to implement machine learning algorithms to devices characterized by a limited computing power and memory [64].

## Figures and Tables

**Table 1:** Comparative table between the scientific work previously analyzed.

Work	N° of Inertial Sensor	Installation Position	Processing	Complexity	Application
P. Janik et al. [42]	1 (LSM9DS1)	Abdominal wall	Digital filtering and peak detection	Low	Breathing and heartbeat monitoring
S. Beck et al. [43]	2 (MPU-6050)	Abdomen and thorax	Digital filtering and frequency domain analysis	Low	Breathing monitoring
M. Hafezi et al. [45]	1	Suprasternal notch	Time domain features extraction and deep learning classifier	High	Breathing monitoring and OSA and hypopnea event detection
C. L. Bucklin et al. [46]	1	Suprasternal notch	Frequency domain analysis, digital filtering, and PCA	Medium	Breathing monitoring and OSA and hypopnea event detection
Doheny et al. [35]	1 (BiostampRC)	Torso	Digital filtering and time domain analysis	Low	Breathing monitoring
T. Eferamawy et al. [48]	2 (LSM9DS0)	Abdomen and thorax	Digital filtering and time domain analysis	Low	Breathing monitoring
J. Vertens et al. [52]	2 (LIS3DSH)	Chest and back	Digital filtering and time domain analysis	Low	Breathing monitoring
S. Lapi et al. [53]	2 (T100)	Sternum	Digital filtering and time domain analysis	Low	Breathing monitoring

**Table 2:** Summarizing table reporting on the characteristics of users that tested and characterized the chest band.

User	Gender	Age (Years)	Height (m)	Weight (Kg)
1	Male	24	1.82	78
2	Male	25	1.89	91
3	Male	25	1.83	82
4	Male	27	1.78	78
5	Female	22	1.71	62
6	Female	23	1.67	56
7	Female	24	1.65	61
8	Female	25	1.70	69

**Table 3:** Parameter sets used to optimize the chest band’s firmware. Set 0 was the starting set.

Parameters Set #	Cut-off Frequency (Hz)	Lag	Threshold	Influence	Minimum Peaks Time Distance (ms)
0	0.5	56	2.95	0.58	1100
1	0.6	52	2.95	0.58	1100
2	0.6	56	2.90	0.58	1100
3	0.6	56	2.85	0.58	1000
4	0.6	58	2.85	0.58	1000
5	0.6	60	2.87	0.58	1000

**Table 4:** Measurements obtained with the various tested parameter sets; in the left column, the measurements provided by the band are reported on, whereas, in the right column, the corresponding reference measurements are shown.

First Set (BrPM)		Second Set (BrPM)		Third Set (BrPM)		Fourth Set (BrPM)		Fifth Set (BrPM)	
Band	Reference	Band	Reference	Band	Reference	Band	Reference	Band	Reference
18	22	20	24	26	26	32	30	26	26
24	24	24	26	26	26	20	20	26	26
20	24	22	24	20	20	30	26	30	30
30	28	22	24	24	24	30	28	30	30
28	28	22	24	20	22	30	30	32	32
24	26	24	24	22	22	28	28	30	30
24	26	24	24	26	24	24	24	30	32
20	20	22	24	20	20	26	26	26	28
20	22	22	22	22	22	26	26	30	30
22	22	20	22	28	26	32	32	26	28

**Table 5:** Summarizing table reporting on the performance of the developed chest band in detecting TI, TE, IER, and V.

Parameter	Pearson Coefficient (r)	MAE (%)
TI (ms)	0.97	4.3
TE (ms)	0.98	3.8
IER (Adim)	0.70	5.0
V (l/min)	0.98	4.3

**Table 6:** Performance comparison of piezoresistive/inertial and differential inertial chest bands considering the user seated, standing, and walking.

	Seated				Standing				Walking			
	$\bar{r}$	$\bar{\mathrm{MD}}$ (BrPM)	$\bar{\mathrm{LoA}}$ (BrPM)	$\bar{\mathrm{MAE}}$ (%)	$\bar{r}$	$\bar{\mathrm{MD}}$ (BrPM)	$\bar{\mathrm{LoA}}$ (BrPM)	$\bar{\mathrm{MAE}}$ (%)	$\bar{r}$	$\bar{\mathrm{MD}}$ (BrPM)	$\bar{\mathrm{LoA}}$ (BrPM)	$\bar{\mathrm{MAE}}$ (%)
Piezoresistive/inertial chest band [28]	0.96	+0.68	+3.20/ −1.75	3.13	0.95	+0.40	+3.34/−2.56	3.56	0.93	+0.23	3.65/ −3.28	3.56
Proposed differential inertial chest band	0.92	−0.27	+1.16/ −1.75	1.15	0.92	+0.56	+3.48/ −2.53	2.69	0.94	+0.48	3.02/ −1.8	3.26

**Table 7:** Comparison of proposed differential inertial chest band with similar breathing measurement systems reported in the scientific literature.

Work	N° of Inertial Sensors	Sensor Position	Processing Unit	Acquisition Frequency (Hz)	$\bar{\mathrm{MAE}}$ (%)	Wireless Connectivity
E. P. Doheny et al. [35]	2 (BiostampRC, MC10 Inc.)	Chest and Abdomen ^(b)^	PC	125	11.13	No
S. Beck et al. [43]	2 (MPU-6050)	Thorax and Back ^(c)^	Arduino MKR1010	N.A. ^(a)^	0.83	No
J. Lee et al. [57]	1 (Biopac MP 150TM)	Chest ^(b)^	PC	500	4.42	No
S. Hughes et al. [58]	1 (Biopac TSD109C2)	Clavicular ^(b)^	PC	500	13.33	No
F. Jacobs et al. [59]	1 (Philips Healthdot)	Lower Rib ^(c)^	Healthdot	100	<25	LoRa
D. Jarchi et al. [60]	1	Chest ^(c)^	PC	25	4.28	No
J. Hernandez et al. [61]	1 (Google Glass)	Head ^(c)^	N.A. ^(a)^	256	10.57	WiFi/BLE
Proposed chest band	2 (MPU-6050)	Chest and Back ^(d)^	SAMD21G18A	100	1.15	BLE 5.0

^(a)^ Not Available; ^(b)^ User Position: Supine; ^(c)^ User Position: Standing; ^(d)^ User Position: Seated.

## Data Availability

Data of our study are available upon request.
